# Acceptance of sexual attraction and its link to psychological distress and sexual offending among pedohebephilic clients: results from a preliminary analysis

**DOI:** 10.3389/fpsyg.2024.1463191

**Published:** 2025-01-27

**Authors:** Anna Konrad, Laura Maria Heid, Hannah Scheuermann, Klaus Michael Beier, Till Amelung

**Affiliations:** Institute of Sexology and Sexual Medicine, Charite - Universitätsmedizin Berlin, Berlin, Germany

**Keywords:** pedophilia, hebephilia, acceptance of sexual preference, sexual offending behavior, distress, Dunkelfeld

## Abstract

**Introduction:**

Pedohebephilic disorder is characterized by intense sexual urges or fantasies involving children, which can lead to distress or sexual behavior with children. While theoretical and qualitative accounts suggest that accepting one’s pedohebephilic sexual interests may help mitigate both distress and problematic behaviors, the only published quantitative study to date has linked acceptance with behavior but did not analyze its effect on distress.

**Methods:**

We examined the relationship between acceptance of sexual interests and child sexual abuse (CSA), the use of child sexual exploitation material (CSEM), and psychological distress in 238 pedohebephilic and teleiophilic men outside the judicial system (i.e., in the “Dunkelfeld”).

**Results:**

Compared to teleiophilic individuals, pedohebephilic individuals showed lower acceptance of their sexual interests. No significant differences were found between groups regarding past sexual offending. In a subsample of 197 pedohebephilic individuals (*n* = 197), correlations with recent sexual behavior were minimal. In another subsample of pedohebephilic men (*n* = 84) with data on psychological distress, increased acceptance was associated with decreased psychological distress, although this association weakened among those reporting recent offenses.

**Discussion:**

Acceptance of one’s sexual interests is associated with reduced distress in pedohebephilic disorder among non-offending individuals. However, its role among offending individuals remains unclear. Efforts to improve measuring the acceptance of one’s sexual interests and further explore its role in pedohebephilic disorder are warranted.

## Introduction

Representative studies in the general population found a notable percentage of adults exhibiting sexual interest in prepubescent or pubescent children, ranging from 2.3 to 5% among men and 0.2% among women (Bártová et al., 2021; Joyal and Carpentier, 2017). This sexual interest can be classified as pedophilia, which refers to prepubescent children, and hebephilia, which involves early pubescent children (Blanchard et al., 2008). Both phenomena often co-occur, and they are addressed together as pedohebephilia (McPhail and Schmidt, 2023; Stephens et al., 2019). Current diagnostic manuals reserve the clinical diagnosis of a paraphilic disorder to conditions where non-normative sexual interests are associated with marked distress or a risk of harm to others through acting upon said interests (DSM-5-TR; American Psychiatric Association, 2022). Concerning pedohebephilia, this differentiation is of special importance in settings outside the judicial system, the so-called “Dunkelfeld,” where individuals with pedohebephilic urges or fantasies may seek help without necessarily having committed any sexual offenses or having committed sexual offenses that remain unknown to the authorities (Beier et al., 2015b).

### Acceptance, pedohebephilia, and offending behavior

Pedohebephilia has seen scientific and clinical interest primarily due to its association with child sexual offenses, i.e., the behavioral disorder criterion, rather than other clinical implications of the condition. Qualitative studies exploring the distress associated with pedophilic disorder have highlighted acceptance of pedohebephilic sexual interests as a potential factor in reducing distress and managing offense risk (Blagden et al., 2018; Dymond and Duff, 2020; Houtepen et al., 2016; Levenson and Grady, 2019; Stevens and Wood, 2019). Accepting one’s pedohebephilic sexual interests may be especially challenging given their societal stigmatization in comparison to normative sexual interests in adults (also termed ‘teleiophilia’) (Jahnke et al., 2015a; Lehmann et al., 2021).

Acceptance as a psychological construct is defined as experiencing events, feelings, and thoughts fully without attempting to change them. It is also associated with improved self-control over impulsive behaviors (Hayes et al., 1994). This concept is integral to approaches in addiction and Dialectical Behavioral Therapy (DBT), where acceptance facilitates self-regulation and control over urges (Linehan, 1987; Marlatt, 1994). In marginalized groups, such as sexual minorities, acceptance has been shown to positively impact mental health and self-esteem (Ong et al., 2021; Puckett et al., 2016; Vosvick and Stem, 2019). There is also evidence suggesting that stigma may lead to risky behavior in terms of vulnerability to HIV infection in cases of homosexual men (Ha et al., 2015). An assumption not empirically examined yet suggests that acceptance of one’s pedohebephilic preference facilitates sexual and general self-regulation and, thereby, behavioral control (Beier, 2021). Acceptance, thereby, is understood as a state that is malleable therapeutically. According to this assumption, a lack of acceptance of one’s pedohebephilic sexual preference leads to an increased risk of sexual offending against children in terms of child sexual abuse (CSA) or the use of child sexual exploitation materials (CSEM).

Data from qualitative studies endorsed this viewpoint. Interviewees in a qualitative study of 15 Belgian and Dutch individuals with pedohebephilia reported that the acceptance of their feelings helped them to deal with their urges (Houtepen et al., 2016). Three British non-offending pedohebephilic men reported that acceptance of their pedohebephilic preference led to productive management of feelings to avoid offending, whereas repression was seen as a maladaptive strategy (Dymond and Duff, 2020). Blagden et al. (2018) examined the impact of pedohebephilic interest on the psychosexual identity of those affected in a forensic sample of individuals who sexually offended against children in semi-structured interviews of *N* = 20 participants. According to this study, having a sexual interest in children is likely to cause dissonance in a general strive for consistency in one’s identity. The authors concluded that those affected by sexual interest in children needed to accept that their sexual interest may never change, enhance sexual self-regulation, recognize triggers, and manage sexual thoughts, feelings, and behaviors.

A recent quantitative study, however, challenged this assumption and suggested that acceptance might, in contrast, be associated with a higher risk of sexual offending against children (Lampalzer et al., 2021). The authors examined a sample of *N* = 79 self-identified pedohebephilic participants reporting prior CSEM and CSA offenses (92.4% of their sample) or no child sexual offenses at all (7.6% of their sample) self-referring to a treatment program outside the judicial system. They administered the previously unpublished *Inventory for the Acceptance of Sexual Inclination* (IASI) as a measure for acceptance of one’s sexual inclinations. The IASI was uncorrelated with dynamic risk factors for CSA offending as measured by the actuarial risk assessment instrument STABLE-2007 (Hanson et al., 2007; Rettenberger and Eher, 2016) but showed a medium positive association with the frequency of use of legal child imagery (*r* = 0.41) and with the frequency of sexual activities with minors (*r* = 0.30) in the past 6–12 months. The authors thus discussed that acceptance of one’s sexual inclination in minors might be associated with an increased risk of offending. As possible mechanisms, they proposed that a greater awareness of one’s sexual interest in children might lead to a greater “presence” of sexual desire or that less acceptance of it might lead to greater efforts to refrain from associated behaviors. Doubts remain, given the unvalidated measure used and the problems with statistical power discussed by the authors.

### Acceptance, pedophilia, and psychological distress

The above-mentioned considerations around acceptance of one’s pedohebephilic sexual inclinations focus on the behavioral disorder criterion of risk of offending sexually against children rather than the distress criterion. Increased psychological distress, however, is well established for pedohebephilic populations (Beier et al., 2015a; Jahnke et al., 2015b; Konrad et al., 2017), and no significant association was found between psychological distress and sexual offending history (Konrad et al., 2017). For non-offending pedohebephilic individuals, self-acceptance of their sexual inclination has been identified as a central therapeutic goal to cope with shame and stigma (Levenson et al., 2020). Further, individuals with pedohebephilic sexual interests viewed acceptance of their inclinations not only as crucial for their mental health but also for their motivation to control their sexual urges towards minors in the form of achieving pride concerning their management (Jones et al., 2021; Levenson and Grady, 2019; Stevens and Wood, 2019). The relationship between acceptance of one’s pedohebephilic inclination, psychological distress, and offense risk is complex. Qualitative data suggest that acceptance can mitigate distress and reduce the likelihood of offending by promoting motivation for behavioral control. However, the only quantitative study to date (Lampalzer et al., 2021) finds an association between acceptance and increased offense risk, though this study predominantly included individuals with a history of child sexual offenses, unlike qualitative studies, which partly included non-offending individuals. This difference in participant backgrounds suggests that acceptance may have a different relationship with distress depending on whether or not an individual has a history of offending. Further research, especially quantitative studies, is essential to clarify these relationships. The current literature indicates that acceptance might reduce psychological distress and help prevent offenses in non-offending individuals, while offending individuals may show a different pattern. In summary, while qualitative and theoretical work suggests that acceptance could have preventative benefits for both mental health and offense risk, quantitative data present an unclear and potentially opposite picture, warranting more exploration into these underlying associations and the validity of the measures used.

### The present study

The aims of this study were thus to examine the acceptance of one’s sexual interests as a state-like feature in pedohebephilic individuals and its relation with recent psychological distress and recent sexual offending against children. Based on the literature on the stigmatization of pedohebephilia, we phrased our first hypothesis:

*H1*: Individuals with pedohebephilia report less acceptance of their sexual inclinations than individuals without pedohebephilia.

Based on one prior study on the association between recent offending and acceptance of one’s sexual preference, we derived two hypotheses:

*H2*: Individuals with pedohebephilia who recently committed child sexual offenses or used child sexual abuse material show greater acceptance of their sexual inclinations than non-offending individuals.

*H3*: State-like acceptance of one’s sexual inclinations is positively correlated with recent offending behavior in individuals with pedohebephilia

*H3a*: Acceptance of one’s sexual inclinations is positively correlated with the frequency of recent CSA behavior in individuals with pedohebephilia.

*H3b*: Acceptance of one’s sexual inclinations is positively correlated with the frequency of recent use of CSEM in individuals with pedohebephilia.

Note that these are composite hypotheses testing for multiple separable behaviors at once. These hypotheses thus posit that acceptance of one’s sexual inclinations is positively correlated with at least one indicator of offending behavior. Accordingly, the corresponding null hypotheses posit that all such correlations are zero or negative.

Based on findings from qualitative studies, we derived the hypothesis:

*H4*: Acceptance of one’s sexual inclination is negatively associated with psychological distress in individuals with pedohebephilia.

Given the somewhat contradictory findings from qualitative studies involving non-offending individuals and the single quantitative study that included both offending and non-offending individuals regarding the association between acceptance of one’s sexual inclinations and recent offending behavior, we formulated our hypothesis.

*H5*: The association of acceptance of one’s sexual inclination with psychological distress is moderated by offensive behavior.

As the factorial structure of the IASI has not been assessed and the intended factorial structure was not documented, we conducted psychometric analyses and an exploratory factor analysis (see Supplementary material).

## Methods

### Procedure

**Figure 1:**
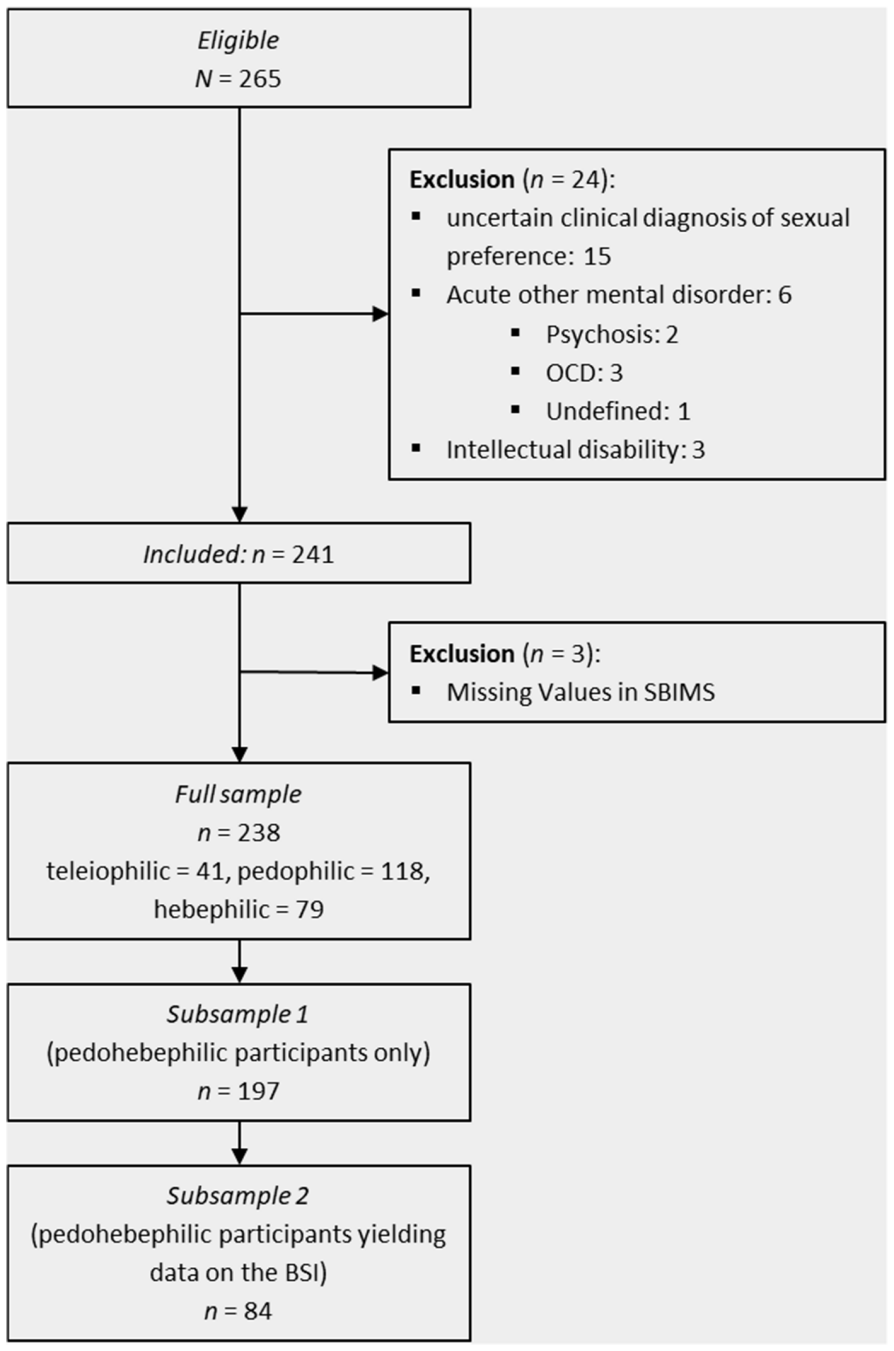
Sample composition.

The present study was conducted within an ongoing project offering diagnostic assessment and therapy to people feeling sexually attracted to prepubescent or early pubescent children. The institution’s institutional review board, which is affiliated with the project, approves all procedures. Data were collected between 2007 and 2014. Participants underwent a semi-structured clinical interview and paper-and-pencil questionnaire testing. All interviewers were trained clinicians with a background in psychiatry, psychosomatics, or psychotherapy with special training for sexual disorders, including paraphilias and sexual offending. All applicants for the project older than 18 years of age and with sufficient German literacy were eligible for the study. Exclusion criteria for this study comprised additional mental disorders with the need for acute treatment (e.g., active psychosis, severe obsessive-compulsive disorder, depression, and suicidality), intellectual disability, uncertain clinical diagnosis of sexual preference, and missing values in questionnaires assessing sexual behavior (see also Figure 1).

Participants were diagnosed with pedophilia or hebephilia based on their self-reported sexual fantasies involving children. A diagnosis of pedophilic disorder was made if the person reported recurrent, intense sexually arousing fantasies or urges involving prepubescent children for at least 6 months, and these fantasies or urges caused clinically significant distress or impairment, or if the individual acted on these fantasies. These criteria are in accordance with those given in the DSM-5-TR (American Psychiatric Association, 2022), with the exception that sexual behaviors with a child without accompanying fantasies or urges did not meet the diagnostic criteria.

Similarly, a hebephilic disorder was diagnosed (categorized as other specified paraphilic disorder) if the participant reported recurrent, intense sexually arousing fantasies or urges involving early pubescent children accompanied by distress, impairment, or actions. If sexual fantasies or urges involved both prepubescent and early pubescent children, pedohebephilic sexual preference was diagnosed. Exclusivity of pedophilic or hebephilic disorder was diagnosed if fantasies involving adults were denied. If sexual fantasies and urges revolved solely around adult persons, a teleiophilic sexual preference was coded. The clinical diagnoses of pedophilia and hebephilia were ascertained in several consecutive steps. Based on the information given in the interview, the clinician gave a suspected diagnosis. A second independent clinician then rated the information documented by the interviewer. This second rating could confirm the first diagnosis (primary agreement). In cases of uncertainties, missing information, or diverging clinical impressions, the interviewer and rater conferred to reach an agreement (secondary agreement). If no agreement was possible, the case was marked as uncertain, and further steps to ascertain the clinical diagnosis were initialized within the context of the treatment program (further examination, third-party anamnesis, or other). Such uncertain cases were removed from the present analysis. Information on primary or secondary agreements was not documented. In summary statistics throughout this paper, groups were constructed reflecting the youngest relevant developmental body schema, i.e., “pedophilic” groups may comprise individuals with exclusive pedophilia, pedohebephilia, and pedohebeteleiophilic sexual preferences.

With the data gathered in an ongoing clinical program, changes in the diagnostic setup of the program led to missing data for some of the analyses (see also under sections “Participants” and “Statistical analyses,” subheading “Association of acceptance with psychological distress…”).

### Participants

The overall sample consisted of *N* = 238 pedophilic (50%, *n* = 118), hebephilic (33%, *n* = 79), and exclusively teleiophilic men (17%, *n* = 41) aged 18 to 81 years (*M* = 37.97, *SD* = 12.27). The reasons for the exclusively teleiophilic men to contact a project for people feeling sexually attracted to children comprised concern about other sexual preferences, sexual boundary violations, or past sexual offending behaviors against children that were not motivated by a sexual preference for children. Pedo-, hebe-, and teleiophilic participants were comparable on most sociodemographic data. Significant group differences emerged in terms of age, gender preference, and recent offending behavior (see Supplementary Appendix A for the description of sociodemographic data). For age, no significant *post-hoc* comparisons emerged, though pedophilic individuals’ mean age was the lowest (36.0 years), whereas hebephilic and teleiophilic individuals’ mean ages were comparable (39.6 and 40.5 years). In the group of teleiophilic men, attraction to female partners was overrepresented, whereas in the group of pedophilic individuals, attraction to females was underrepresented. Additionally, teleiophilic men were overrepresented in the group of non-offending individuals and underrepresented in the group of those who had recently shown CSA-only offending.

Two subsamples were created from our overall sample to facilitate our planned analyses. The first subsample consisted only of pedohebephilic men (*n* = 197). The sociodemographic distribution revealed no significant differences from the overall sample. These individuals ranged in age from 18 to 81 years (*M* = 37.45, *SD* = 12.15). Approximately one-third (31%) of these men reported that their sexual fantasies and urges involved exclusively children and were thus classified as exclusively pedohebephilic. In terms of recent offending behavior, a majority (*n* = 130; 66%) reported offenses involving only CSEM. A smaller proportion, one-sixth (*n* = 31; 16%), reported no recent offending. Moreover, 29 men (15%) admitted to both recent CSA and CSEM offenses (mixed offending), and seven more men (4%) were identified as recent CSA-only offending individuals.

There were no significant differences between the four offening groups regarding their sexual age and gender preference, exclusivity of their sexual preference, or any other collected sociodemographic variables (see Supplementary Appendix A).

The second subsample consisted of *n* = 84 pedohebephilic men aged 18 to 81 years (*M* = 38.26, *SD* = 12.57) who yielded data on the questionnaire measuring psychological distress. Details for inclusion and exclusion can be found in Figure 1.

### Measures

#### Acceptance of one’s sexual inclination

For the present study, we used a modified nine-item version (IASI-9) of the *Inventory for the Acceptance of Sexual Inclination,* IASI (Ahlers et al., 2008; Mundt et al., 2011). The IASI is a self-report instrument to assess the degree of a person’s acceptance of their sexual inclination. The items of the IASI were developed based on statements of help-seeking pedohebephilic clients concerning their sexual inclinations and fantasies. The items were supposed to reflect four apriori defined scales: “Attitude”—one’s attitude towards accepting one’s sexual preference and knowledge of the importance of acceptance; “Perceived Acceptance”—the subjective extent of acceptance; “Emotions”—the emotional processing of the sexual preference as an indicator of the extent of acceptance; and “Fantasies and Control”—handling of sexual fantasies and needs as an indicator of the extent of acceptance. The scale has not yet been validated, and the assignment of the items to the four scales has not been published. The items are to be rated on a 5-point-Likert scale ranging from 1 (*This statement is... not true at all*), 2 (.*..not very true*), 3 (*... moderately true*), 4 (*...quite true*) to 5 (*...very true*). Total scores of the IASI range from 15 to 75. Only one study reported an internal consistency of Cronbach’s α = 0.88 (Lampalzer et al., 2021). The internal consistency for the sum score of the 15 items within our overall sample was similar to Lampalzer et al. (α = 0.88, 95% CI [0.86, 0.90]).

As the factorial structure has not yet been assessed and the intended factorial structure was not documented, we conducted psychometric analyses and an exploratory factor analysis (see Supplementary Appendix C). The analyses revealed poor psychometric properties such as high means and left-skewed distributions (items 12 and 13) as well as low discriminatory power of a number of items (items 5, 10, 12, 13, and 14, see Supplementary Material Figure C1) and suggested a two-factor solution as the most parsimonious. In this solution, psychometrically unfavorable items (5, 12, 13, and 14) loaded together on one of the factors (Cronbach’s α = 0.47, 95% CI [0.35, 0.57]), all exhibiting low communalities (see Supplementary Table C5). Furthermore, one item showed a complex loading (8), and one did not load significantly on either factor (10). Given the psychometrical problems and the low communalities, these items, as well as the complex and not loading items, were excluded from further analyses.

The other factor comprised nine items with favorable distributional properties that showed excellent internal consistency (Cronbach’s α = 0.92, 95% CI [0.90, 0.93]) and was theoretically meaningful in that all items reflected statements surrounding the rejection or acceptance of one’s sexual inclinations or fantasies. As items reflecting the rejection of one’s sexual inclinations or fantasies were inverted for this study, we labeled this factor “acceptance of one’s sexual inclinations and fantasies,” or “acceptance,” and the suggested reduced scale IASI-9. Details on the distribution of the items and the overall scale can be found in Table 1.

**Table 1:** Descriptive statistics of the retained factor of the IASI-9, the individual items loading on the retained factor, and the GSI in the full sample, subsample 1, and subsample 2.

Variables	Full sample (*N* = 238)						Subsample 1 (*n* = 197)		Subsample 2 (*n* = 84)	
	*Mdn*	Range	Skew	Kurtosis	Difficulty (*P*_i_)	Discriminatory power *R*_it,c_	*Mdn*	Range	*Mdn*	Range
IASI-9 sum score	23	9–45	0.37	−0.9	–	–	22	9–45	21	9–45
Item 3: I hate my sexual inclination^a^	2	1–5	0.54	−1.20	35.92	0.71	2	1–5	2	1–5
Item 6: I reject my sexual inclination^a^	2	1–5	0.48	−1.29	37.82	0.74	2	1–5	2	1–5
Item 4: I cannot accept my sexual inclination as^a^	2	1–5	0.39	−1.25	40.23	0.76	2	1–5	2	1–5
Item 11: My sexual fantasies scare me^a^	2	1–5	0.42	−1.09	39.39	0.68	2	1–5	2	1–5
Item 9: I reject myself because I have this sexual inclination toward^a^	3	1–5	−0.01	−1.36	52.82	0.61	3	1–5	3	1–5
Item 2: I can enjoy my sexual fantasies without a bad conscience	2	1–5	0.50	−0.84	37.29	0.72	2	1–5	2	1–5
Item 15: I resist my sexual fantasies^a^	3	1–5	−0.21	−1.10	54.41	0.72	3	1–5	3	1–5
Item 1: I forbid myself my sexual fantasies^a^	3	1–5	−0.28	−0.96	58.72	0.64	3	1–5	3	1–5
Item 7: One must resist disagreeable sexual fantasies^a^	2	1–5	0.49	−1.09	38.66	0.56	2	1–5	2	1–5
GSI (*M*, *SD*)	–	–	–	–	–	–	–	–	1.02	0.57

IASI-9, Nine-item version retained after exploratory factor analysis (see Supplementary material C for details) of the Inventory for the Acceptance of Sexual Preference. GSI, Global Severity Index of overall psychological impairment from the Brief Symptom Inventory (BSI). Discriminatory power was determined as a corrected item-scale correlation. Subsample 1 included only pedohebephilic participants of the full sample. Subsample 2 included only pedohebephilic participants in the full sample, providing data on the BSI. Items were inverted.

#### Psychological distress

The *Brief Symptom Inventory* BSI (Derogatis, 1993), German version (Franke, 2000), is a short version of the *Symptom Checklist-90-Revised*, SCL-90-R (Derogatis, 1977). It measures self-reported psychological symptoms within the past 7 days. With 53 items, it covers the same nine symptom dimensions as the SCL-R-90: somatization, obsession-compulsion, interpersonal sensitivity, depression, anxiety, hostility, phobic anxiety, paranoid ideation, and psychoticism. Items (e.g., “Your feelings being easily hurt”) are to be answered on a 5-point Likert scale ranging from 0 (*not at all*) to 4 (*extremely*). Scores can be calculated for each dimension as well as for three global indices: (1) The Global Severity Index (GSI), (2) The Positive Symptom Total (PST), and (3) The Positive Symptom Distress Index (PSDI). In this study, we used the GSI as the most sensitive indicator of the respondent’s overall psychological impairment (i.e., distress level). The author of the BSI reported good test–retest reliability for the GSI (0.90) and high correlations between the dimensions of the BSI and SCL-R-90 in a sample of US psychiatric outpatients (0.92–0.99). In German samples, the internal consistency of the GSI ranged from α = 0.92–0.96 (Franke, 2000). In the sample used in our study, Cronbach’s alpha was *α* = 0.95.

The GSI is a composite measure indicating the level of symptomatology, which is calculated as the mean of all items ranging from 0 to 4, with higher values indicating higher levels of psychological distress. Scores are generally interpreted by comparison to age-appropriate norms. Based on different populations, a GSI value of 0.62 (raw score of 33, normalized *t* score of 63) has been proposed as the cut-off for clinically significant global distress (Derogatis, 1993). The German manual lists mean GSI for normal male controls (*N* = 300, *M* = 0.28, *SD* = 0.23), psychiatric male inpatients from the US (*N* = 158, *M* = 0.97, *SD* = 0.78), and psychiatric male outpatients from the US (*N* = 425, *M* = 1.20, *SD* = 0.70).

#### Behavioral measures

##### Recent sexual offense behavior

The *Sexual Behavior Involving Minors Scale*, SBIMS (Neutze et al., 2011), is an unpublished 8-item self-report measure assessing the frequency of sexual behaviors with children in the past 6 months by asking for masturbation frequency including child sexual fantasies (4 items, Cronbach’s *α* = 0.76. 95% CI [0.71, 0.82] in subsample 1), frequency of the use of CSEM (1 item), and frequency of sexual behaviors with children including non-physical sexual interactions, sexual activities in the presence of a minor, and physical contacts with a minor (3 items, Cronbach’s α = 0.80, 95% CI [0.75, 0.85] in subsample 1). Items are based on German jurisdiction concerning child sexual offending. The item assessing physical contact is phrased slightly ambiguously (see Supplementary Table B2). A sensitivity analysis assessed the influence of this item on the results (see Supplementary Appendix E). Higher scores indicate a higher frequency of these behaviors. Items are rated on a 5-point Likert scale, anchored as 1—“*never*,” 2—“*on few occasions*,” 3—“*monthly*,” 4—“*weekly*,” and 5—“*daily*.” Values for recent CSA behaviors range from 3 to 15. Recent CSA behaviors were coded dichotomously for values ≥4.

The *Questionnaire for Sexually Explicit and Non-Explicit Images of Children and Adults* (Q-SENICA) is an unpublished 30-item questionnaire assessing the use of sexually explicit and non-explicit images for sexual arousal, including CSEM, in the past 6 months. Items assess the use of different materials on a scale anchored as 1—“*never*,” 2—“*on few occasions*,” 3—“*monthly*,” 4—“*weekly*,” and 5—“*daily*.” Items were developed from patients’ statements. Materials assessed include indicative material (depictions of clothed minors from magazines, movies, and so on), erotic posing (minors dressed in tight or erotic clothing or posing nude), explicit posing (depictions focused on minors’ genitals or involving self-touching), and sexual assault (genital interactions including penetrative acts; for translated items, see Supplementary Table B1). Separate items ask for the use of materials for masturbation and pastime to avoid underreporting due to self-serving bias. From all three different types of CSEM, a single dichotomous variable for recent CSEM offending or recent CSEM non-offending individuals was summarized (Cronbachs *α* = 0.96).

The variables for recent CSA (SBIMS) and CSEM offending (Q-SENICA) were then combined into (a) a dichotomous variable representing recent offending (CSA and/or CSEM) vs. recent non-offending individuals (neither CSA nor CSEM) and (b) a categorical variable, which classified the recent offense status of the participants into four categories: recent non-offending, recent CSEM-only offending, recent CSA-only offending, and recent mixed offending. German law concerning CSEM changed since the inception of this study. The phrasing of the items of the Q-SENICA assessing erotic posing encompasses aspects that, under the current jurisdiction, may count as either legal or illegal imagery. For this study’s group comparison and regression analyses, we classified said items as reflecting illegal behavior. To assess the influence of this pre-analytic decision, we added a sensitivity analysis (see Supplementary Appendix D) in which we classified these items as reflecting legal imagery.

##### Frequency of recent sexual offense behavior and desire

*The Questionnaire on Sexual Experiences and Behavior* (Q-SEB) (Ahlers et al., 2004) is a standardized unpublished paper-and-pencil questionnaire assessing sexually relevant information on essential areas of human sexual experience and behavior with 11 modular scales (e.g., sexual fantasies, gender role identification, sexual preference, and sexual offenses). Only items of the subscale *Sexual Activity* were used, which assess sexual activity and the desire for sexual activities in a timeframe of the past 12 months. Items are to be answered on an 8-point Likert scale ranging from 1 (*never*) to 8 (*once or multiple times daily*).

##### Aggregated variables of recent sexual offense behavior

To assess the frequency of recent sexual offense behavior, we built variables paralleling those of Lampalzer et al. (2021): (1) frequency of use of legal imagery of children, (2) frequency of use of legal imagery of adolescents, (3) frequency of use of illegal imagery of children, (4) frequency of use of illegal imagery of adolescents, (5) frequency of desire for sexual activities with minors, (6) frequency of sexual activities with minors. The variables were built by taking the value of the highest frequency of the relevant items from the Q-SENICA, SBIMS, and Q-SEB (see Supplementary Table B2 for the specific questions used from Q-SENICA, SBIMS, and Q-SEB for each variable). Since the Q-SEB assesses desire for sexual activities with minors within the past year, we computed two variables assessing the *Frequency of Sexual Activities with Minors*: One covering the past 6 months based on items from the SBIMS (item 6a) and one covering the past 12 months based on items from the Q-SEB (item 6b). Note that illegal behaviors were defined slightly differently for these frequency variables and the allocation to a group based on recent sexual offending behavior. This concession was made to increase comparability with the results of Lampalzer et al. (2021). For the variables assessing the frequency of use of illegal imagery, we excluded images of lightly dressed children and adolescents (e.g., in underwear and gym shorts) and images of interaction between children and adolescents, paralleling the construction in Lampalzer et al. (2021). However, for the allocation to the groups of recent use of CSEM, both categories of images were taken into account in accordance with the German criminal code. The pattern of the inter-item correlations of the six variables assessing the frequency of use of abuse/exploitation material of minors and frequency of sexual desire/behavior towards minors is given in Supplementary Table B3.

### Statistical analyses

All statistical analyses were conducted using *RStudio* for macOS (2022.07.2 + 576 “Spotted Wakerobin” © 2009–2020 RStudio, PBC, R 4.2.2 “Innocent and Trusting” © 2022). The R Foundation for Statistical Computing.

#### Group comparisons

There are no published effect sizes for these comparisons, impeding a sample size estimate. Data from the IASI was mostly non-normally distributed within the groups. We thus employed the Kruskal-Wallis test for group comparisons of the IASI. Differences in acceptance of sexual inclinations and fantasies between groups separated by sexual preference were examined within the overall sample of *N* = 238. Differences between offending groups were examined in the subsample of *n* = 197 without exclusively teleiophilic individuals to avoid interference with sexual preference. We followed up on significant effects in groups separated by sexual preference and by type of offending, respectively, using Dunn’s test with Benjamini and Hochberg’s (1995) adjustment of the *p-*value for multiple testing. Effect sizes for the pairwise *post hoc* comparisons were computed as *rho* and interpreted following Cohen’s rules of thumb.

#### Association between acceptance and frequency of recent behavioral manifestations and sexual desire

To estimate the sample size required for a reproduction of the prior study using the programs G*Power (Faul et al., 2013) and FDRSamplesize2 (Ni et al., 2024), we identified the smallest correlation reported in Lampalzer et al. (2021), pertaining to our hypotheses H3 through H3b, i.e., desire for sexual activities with minors, *r* = 0.234. For an uncorrected test with *α* = 0.05 and 1 – *β* = 0.80, a minimum sample size of 111 was needed to replicate this correlation. Considering a correction for multiple comparisons using the Benjamini-Hochberg correction with an FDR = 0.05 and 1–*β* = 0.80, this number rose to 148. We assessed the relation between acceptance of one’s sexual inclinations and fantasies and the frequency of behavioral manifestations and sexual desire towards minors in the subsample of 197 without exclusively teleiophilic individuals.

We applied Spearman’s *rho* with the Benjamini-Hochberg correction (Benjamini and Hochberg, 1995) to account for multiple testing across seven tested correlations. This decision was made in light of the broad research question concerning the potential relationship between acceptance of one’s sexual inclinations and the frequency of recent sexual behavior and desires towards minors. Spearman’s rho coefficients were interpreted following Cohen’s guidelines (1988).

#### Association of acceptance with psychological distress and recent offending behavior

Changes in the diagnostic setup of the running program led to missing data for the BSI in several study participants. The regression analyses were thus conducted in Subsample 2 of pedohebephilic individuals, which provided data on the BSI (*n* = 84). We applied a multiple linear regression model with acceptance of sexual inclinations and fantasies as the dependent variable and recent offending behavior and psychological distress as independent variables. The model included interaction terms to test whether the association between acceptance and the predictors varied based on interactions between offending and distress. No effect sizes have been published to estimate the sample size needed for this analysis. We analyzed standardized residuals using deviance from the expected distribution and absolute values greater than 3 as cause for concern (Field, 2013). A hat value greater than three times the average leverage was set to identify cases with undue influence (Pituch and Stevens, 2016). We further used Cook’s Distance greater than 1 to identify influential cases (Cook and Weisberg, 1982), Watson Durbin test statistic close to 2 to test the assumption of independent errors, variance inflation factors (VIF, in models with interactions in the form of $GVIF^{\frac{1}{2*df}}$ ) less than or equal to 10 to assess multicollinearity, and a scale-location plot and a Breusch-Bagan test to assess equal variance of error (Field, 2013). The assumption of linearity and normal distribution of residuals were checked visually via a scatterplot of residuals vs. fitted values and a Q-Q plot and histogram, respectively.

Our first hierarchical analysis regressed acceptance of recent offending behavior (CSA and/or CSEM) and distress measured by the *Global Severity Index*, GSI, of the BSI. In a second step, we entered the interaction term of the dichotomous offense variable and the GSI score into the model to investigate whether the association of psychological distress and acceptance might be moderated by recent offending behavior.

We further examined significant interactions using a simple slope analysis (Aiken and West, 1991). Common support for interaction terms (i.e., the variation of regressor scores for a particular value of the moderator) (Hainmueller et al., 2019) was assessed visually via a scatterplot of the dependent variable by regressor with dots colored according to the moderator category.

## Results

### Group comparisons of acceptance and correlations with recent offending behavior and desire (hypotheses H1 through H3b)

Acceptance of their sexual inclinations and fantasies differed significantly between the preference groups [*H*(2) = 21.94, *p* < 0.001]. Specifically, teleiophilic individuals reported significantly higher acceptance (Mdn = 37, range = 10–45) than both pedophilic (Mdn = 22, range = 9–45, *p* < 0.001, *rho* = 0.29) and hebephilic individuals (Mdn = 22, range = 10–44, *p* < 0.001, *rho* = 0.27). For H1, the null hypothesis was thus rejected. Pedophilic and hebephilic men’s acceptance of their sexual inclinations and fantasies did not differ significantly (*p* = 0.923, *rho* = 0.006). No significant differences in acceptance were observed between non-offending individuals (Mdn = 22, range = 9–45), those who offended exclusively via child sexual exploitation material (CSEM; Mdn = 22, range = 9–45), those with child sexual abuse (CSA)-only offenses (Mdn = 22, range = 18–44), and those with mixed offending behavior [Mdn = 22, range = 10–44; H(3) = 5.98, *p* = 0.113]. Sensitivity analysis found no influence of the ambiguous questionnaire items concerning CSAI or CSA behavior on this outcome (see Supplementary Appendices D, E). For H2, the null hypothesis thus could not be rejected.

Correlations with variables reflecting desire for sexual activities with children in the past 12 and frequency of use of imagery depicting children in the past 6 months were positive but smaller than *rho* = 0.1 (see Table 2). Acceptance showed a small positive correlation with the self-reported frequency of sexual activities with minors within the past six (*rho* = 0.177) and 12 months (*rho* = 0.136), the former being slightly larger and reaching significance when omitting the ambiguous item concerning physical contact with minors (see Supplementary Appendix E). None of the other correlations were significant after the Benjamini-Hochberg correction (see Table 2). Support for hypotheses H3 and H3a was thus ambiguous, with the effect depending on the operationalization of CSA offending, whereas hypothesis H3b was not supported.

**Table 2:** Descriptive statistics and correlations of the IASI-9 and measures of recent sexual behavior and desire.

Variable	*Mdn*	Range	*rho*	FDR adjusted *p*_BH_	*n*
Frequency of…					
(1) … Use of Legal Imagery of Children	2	1–5	0.039	0.806	197
(2) … Use of Legal Imagery of Adolescents	2	1–5	0.016	0.821	197
(3) ….Use of Illegal Child Sexual Abuse Imagery	2	1–5	−0.039	0.806	197
(4) … Use of Illegal Adolescent Sexual Abuse Imagery	3	1–5	−0.029	0.806	197
(5) …Desire for Sexual Activities with Minors	4	1–8	0.091	0.480	193
(6a) … Sexual Activities with Minors (SBIMS)	1	1–5	0.177	0.088	197
(6b) … Sexual Activities with Minors (Q-SEB)	1	1–8	0.136	0.205	193

Correlation coefficients are computed as Spearman’s rho. (1)–(4), (6a): Frequency within the past 6 months. (5), (6b): Frequency within the past 12 months. IASI-9 = Nine-item version retained after exploratory factor analysis (see Supplementary material C for details) of the Inventory for the Acceptance of Sexual Preference. 6a. Amalgamated items of the SBIMS to assess the frequency of sexual activity with minors. 6b Amalgamated items of the Q-SEB to assess the frequency of sexual activity with minors.

### Association of acceptance with psychological distress and recent offending behavior (hypotheses 4 and 5)

In our second subsample of *n* = 84 individuals with pedohebephilic sexual interests providing data on distress, 60 men (71%) reported clinically significant levels of global psychological distress with a GSI ≥ 0.62.

The hierarchical regression analysis model of acceptance of one’s sexual inclinations and fantasies on general recent offending behavior and psychological distress without interaction terms was statistically significant [*F*(2, 81) = 7.02, *p* = 0.002] and explained 15% of the variance (Table 3). Psychological distress was significantly and negatively associated with acceptance (*p* < 0.001), i.e., as psychological distress increased, acceptance of one’s sexual inclination decreased. In our main and sensitivity analyses, recent general offending behavior was not significantly associated with acceptance (*p* = 0.625).

**Table 3:** Hierarchical regression of acceptance of sexual inclinations and fantasies on general recent offense behavior and psychological distress.

Coefficients	Step 1 (without interaction)				Step 2 (with interaction)			
		95% CI				95% CI		
	*b*	*LL*	*UL*	*SE*	*b*	*LL*	*UL*	*SE*
Constant	27.28***	23.01	31.55	2.15	32.79***	47.68	63.93	3.19
Offending1	1.02	−3.13	5.17	2.09	−6.20	−15.43	3.71	3.75
GSI	−5.60***	−8.59	−2.61	1.50	−12.73***	−23.24	−5.71	3.44
Offending1 x GSI	–	–	–	–	8.71*	−0.001	19.37	3.80
*R* ^2^				0.15**				0.20***
Adjusted *R*^2^				0.13				0.17

Dependent variable: Sum score of the IASI-9 “Acceptance of one’s sexual fantasies and inclinations.” The offender variable is coded as 0 = Non-Offending and 1 = Offending. GSI = Global Severity Index of the Brief Symptom Inventory. *p < 0.05, **p < 0.01, ***p < 0.001.

The model, including the interaction term, was statistically significant [*F*(3, 80) = 6.67, *p* < 0.001], explaining 20% of the variance of acceptance. Adding the interaction term significantly improved the fit of the regression model [*F*(1, 80) = 5.25, *p* = 0.025]. The estimators for psychological distress (*p* < 0.001) and its interaction with recent offending behavior (*p* = 0.025) were significantly different from zero, while the estimator for recent offending behavior alone was not (*p* = 0.102), i.e., psychological distress and its interaction with recent offending behavior had a significant effect, while recent offending behavior alone did not show a strong effect. When considering the interaction, higher psychological distress was linked to lower acceptance of one’s sexual inclinations and fantasies in non-offending individuals. As indicated by the positive interaction estimator, however, this link was weaker in those who had recently committed CSA or CSEM offenses. Classifying the responses on the item concerning physical contact with minors as no CSA had no influence on the results. Classifying the use of images of lightly dressed children as legal behavior yielded a slight increase in all estimates, including a significant negative association of offending behavior with acceptance (*b* = −7.91, *SE* = 3.53, *p* = 0.028). The signs of the estimators remained unchanged. All assumptions about outliers, influential cases, independent errors, multicollinearity, equal variance of error, linearity, and normal distribution of residuals were met.

**Figure 2:**
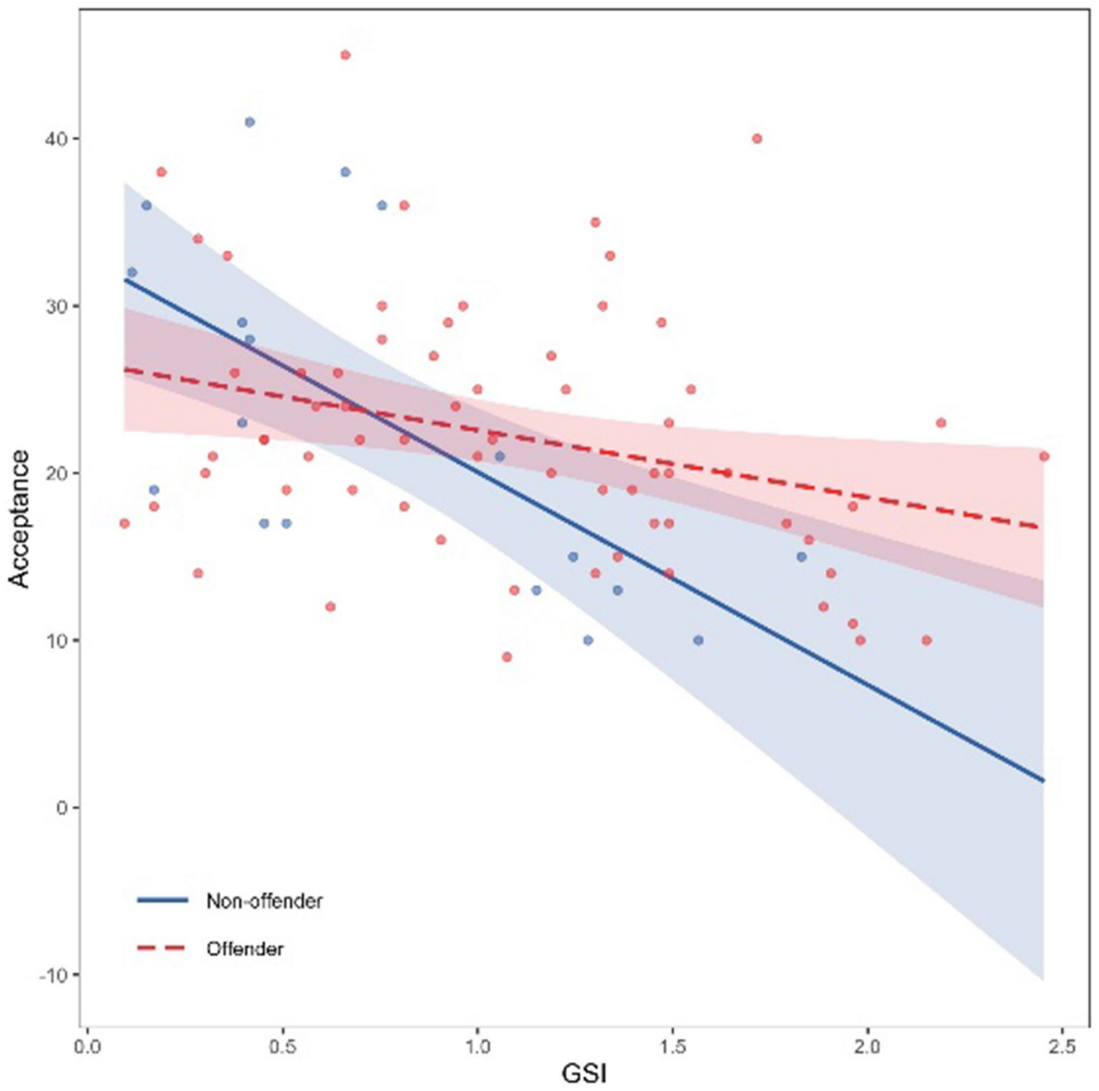
Association of the interaction between recent offending behavior and psychological distress with acceptance of one’s sexual inclinations and fantasies. Acceptance of sexual inclinations and fantasies measured by the IASI-9. GSI = Global Severity Index of the *Brief Symptom Inventory*. The theoretical range of the mean GSI is 0–4. Maximum values in this study reached 2.45.

Common support for the interaction between distress and recent offending behavior was acceptable (Figure 2). The simple slope of the association of distress with acceptance for recent offending behavior differed significantly from zero (*b* = −4.02, 95%-CI [−7.24, −0.80], *p* = 0.015), as did the simple slope of the association in recent non-offenders (*b =* −12.73, *p* < 0.001). Thus, in both groups, there was a negative association between distress and acceptance, which was stronger in recent non-offending than in recent offending individuals.

## Discussion

### Group comparisons of acceptance of one’s sexual preference

Our study is the first to compare the acceptance of sexual inclinations and fantasies between pedohebephilic and teleiophilic individuals. The non-teleiophilic individuals reported lower acceptance of their sexual preference than teleiophilic individuals.

This finding should be interpreted cautiously, as measurement invariance between the groups has not yet been established. It is expected that acceptance levels would be lower among individuals with sexual interests in minors, given the significant social stigma attached to such inclinations. Indeed, qualitative studies have documented that individuals with a sexual interest in minors often face challenges in accepting their sexual inclinations and fantasies (Jones et al., 2021; Stevens and Wood, 2019).

Notably, in our study, the groups differentiated by past offending did not show significant differences in their reported acceptance of sexual inclinations and fantasies, suggesting that reduced acceptance may not be heavily influenced by overt sexual behaviors among this clinical sample of men with pedohebephilia.

### Association between acceptance of sexual inclinations and fantasies and the frequency of recent sexual behavior and desire

Concerning a possible association between acceptance of one’s sexual inclinations and the frequency of fantasies and recent sexual behavior, our study found only small positive correlations. For example, all correlations between acceptance and the frequency of use of legal and illegal imagery of children or adolescents in the past 6 months were smaller than *r* = 0.1. The correlations with the frequencies of sexual activities with minors and desire for such activities in the past 6 or 12 months were slightly larger. However, this difference may not be meaningful given the small sample size and associated imprecision of the estimate. The only prior study on this topic found correlations of magnitudes up to 0.304 between acceptance and the self-reported frequency of sexual activities with minors in the past 12 months (Lampalzer et al., 2021). Comparing their and our correlation coefficients statistically yields no significant difference; e.g., for sexual contact with minors in the last 6 months, a comparison using Fisher’s *z* transformation yields test statistics for the difference of *z* = 0.979, *p* = 0.328. Applying an equivalence testing logic, the lower bounds of the 90%-CIs of our coefficients are smaller (0.06 and 0.01) than those of the 95%-CI of the coefficient in Lampalzer et al. (0.08). In summary, whether or not our results are different from those of Lampalzer et al. (2021) thus remains unresolved, also given both studies relied on rather small sample sizes.

The slightly larger numerical effect in Lampalzer et al. (2021) may be due to a difference in sample composition. This difference becomes apparent on inspection of the pattern of the inter-item correlations of the six variables assessing the frequency of use of sexual exploitation material of minors, sexual desire, and sexual behavior towards minors (cf. Supplementary Table B3). While the overall correlations were similar to Lampalzer et al. (2021), contrasting their findings, the frequency of contact sexual behavior with children (items 6a and 6b) in our sample did not correlate with the frequency of use of legal and illegal imagery of adolescents and children (items 1–4). Also, differing from their findings, the frequency of sexual activities with minors in the past 6 months (item 6a) did not correlate with the frequency of desire for sexual activities with minors (item 5) in our sample. Thus, the patterns of recent sexual offending against children differed between their sample and our sample, with a greater correlation between recent contact with sexual behavior with children and the use of legal and illegal imagery of minors in the other study. Accordingly, the sample employed in the Lampalzer et al. study may have comprised a larger number of individuals who had committed both CSA and CSEM offenses within the past six to twelve months than were present in our sample. A corroboration of this interpretation with actual numbers is impossible, as Lampalzer et al. only gave numbers for lifetime sexual offenses but not recent sexual offending. Either way, the numerical difference in correlations between their and our findings could also be evidence for a moderating effect of recent child sexual offending on the association of acceptance of one’s sexual inclinations and fantasies and different domains of sexual experiences and behavior. For example, a larger association may be present in individuals with combined recent CSA and CSEM offenses or in individuals where the frequency of recent CSA and CSEM offenses correlates more strongly. Interestingly, an association of acceptance of one’s sexual inclinations and fantasies with both legal and illegal behavior is also what Lampalzer et al. (2021) suggest following their analyses.

### The association of acceptance of sexual inclinations and fantasies with offending and distress

Clinically significant levels of global psychological distress were highly prevalent in our pedohebephilic sample. Our hierarchical regression analyses revealed an association between higher levels of psychological distress and lower levels of acceptance of one’s sexual inclinations and fantasies. This association is plausible in the context of societal and self-stigmatization of individuals with a sexual interest in minors (Jahnke et al., 2015a; Lehmann et al., 2021). Being faced with persistent, intense sexual fantasies or urges that one‘s surroundings stigmatize may lead both to problems in accepting the existence of such sexual emotions and to distress, given their persistence. The finding also fits with qualitative studies on mental health issues in individuals with sexual interests in minors, where affected individuals reported that coming to terms with their sexual preference and accepting their attraction had an important impact on their psychological wellbeing (Jones et al., 2021; Stevens and Wood, 2019). Similar effects have been shown for other non-heteronormative sexualities, too (Su et al., 2016). Our finding might thus hint at the contribution of minority stress to the mental health of individuals with a sexual preference for children. In this context, validated measures aiming to address acceptance of one’s sexuality have been published for use in other sexual minorities since the inception of this study, like the Lesbian, Gay, and Bisexual Identity Scale, LGBIS (Mohr and Fassinger, 2000; Mohr and Kendra, 2011; Niepel et al., 2019), or the Self-Acceptance of Sexuality Inventory, SASI (Camp et al., 2022). Both measures are rooted in theories of non-heterosexual identity development in a heteronormative world, which might share similarities to that of pedohebephilic individuals but also arguably will show vast differences, e.g., concerning the opportunities to explore one’s sexuality with consenting others. The applicability of both LGBIS and SASI in samples of individuals with pedohebephilic sexual interests has not been examined yet and appears questionable, at least for the LGBIS, which relies heavily on wordings specific to LGB-identity. The SASI, on the other hand, applies a broader framework for sexuality. From face validity, both the SASI and the IASI items retained for this analysis share similarities, e.g., the SASI item 8: “I try to fight my sexuality” and the IASI items 6: “I reject my sexual inclination” and 15: “I resist my sexual fantasies,” or the SASI item 6: “I struggle to accept my sexuality” and the IASI item 4: “I cannot accept my sexual inclination.” Future endeavors might examine the convergent validity of both measures.

The examination of sexual behaviors revealed an interaction of the association of acceptance of one’s sexual inclinations and fantasies and psychological wellbeing with recent offending behavior. Specifically, the above-mentioned association of acceptance of one’s sexual inclinations and fantasies and psychological distress was significantly attenuated in individuals who had committed any offenses recently. The attenuated association of psychological distress and acceptance of one’s pedohebephilic inclinations and fantasies in offending individuals cannot be readily explained by our study. Several hypotheses to explain this differential come to mind. Offending compared to non-offending individuals exhibit more antisocial traits both in samples of persons convicted for sexual offending and in individuals self-reporting sexual offending in the Dunkelfeld and may thus feel less remorse or less distress because of societal stigmatization (Babchishin et al., 2015; Cantor and McPhail, 2016; Gerwinn et al., 2018; Mann et al., 2010). The missing association may also result from post-hoc neutralizations after a committed offense and associated offense-supportive attitudes (Blake and Gannon, 2008; Helmus et al., 2013). In this line of argument, rationalizations like “I did not do any harm” or “The child wanted it that way” may help reduce the need to negate sexual attractions and thus increase acceptance. The analysis of such associations, however, was beyond the scope of this study and will have to be explored in the future.

In addition to the observations made in the discussion of our correlations analyses above, this finding can be seen as another hint at a moderating effect of recent sexual offending on the clinical relevance of acceptance of one’s pedophilic inclinations and fantasies. From a clinical point of view, acceptance may be a more relevant treatment target for non-offending individuals with pedohebephilia as it may reduce perceived distress in this population, but less so for offending individuals with pedohebephilia. Conversely, as suggested by Lampalzer et al., increased acceptance of one’s pedohebephilic inclinations and fantasies in this population might be associated with a greater risk of sexual offense. So far, however, there is only limited support for this assumption. As suggested by qualitative studies, there could even be a beneficial effect in that acceptance reduces distress and, thereby, the likelihood of offending by promoting motivation for behavioral control.

The interaction effect in our data indicates that psychological distress is associated with lower acceptance of sexual inclinations in non-offending individuals, while this link is less evident in those who have recently offended. This finding highlights the potential value of a clinically differentiated approach to managing pedohebephilic disorder. Such an approach aligns with the widely accepted Risk-Need-Responsivity (RNR) Model for treating individuals who have sexually offended, which cautions against a one-size-fits-all strategy. Instead, the RNR model emphasizes tailoring interventions to individuals’ specific needs and risk levels, as applying uniform methods across groups with differing re-offense risks may lead to unintended harm (Bonta and Andrews, 2007). Slightly diverging from this principle, which states that persons with a high risk of sexual offending will need a greater amount of intervention than those with a low risk of offending, interventions addressing acceptance might be of greater relevance for those deemed to be of lower risk, i.e., non-offending pedohebephilic individuals.

### Limitations

Our choice of sample limits the generalizability of our findings to clinical populations. Importantly, our findings were established in a sample from the Dunkelfeld, i.e., consisting of self-referring, help-seeking individuals outside the judicial system. The generalizability of samples of convicted offending individuals remains unclear. Also, the size of groups differentiated by CSA and CSEM offending impeded further differential analyses. Furthermore, the necessity to build sub-samples based on the available information has reduced the sample sizes, especially for the regression analysis. While a *post-hoc* power analysis revealed a power of (1–*β*) = 0.97 to detect an *R^2^* = 0.2 and of (1–*β*) = 0.96 to detect an estimator *b* = −12.73 for distress with an *α* = 0.05, this power dropped to (1–*β*) = 0.38 for the estimate for offending and (1–*β*) = 0.63 for the interaction. Such a lack of statistical power can lead to both an underestimation of true effects and an overestimation of false effects, and the results need to be viewed as preliminary (Ioannidis, 2005). The fact that our teleiophilic control group consisted of a clinical sample, too, can be seen as further support of our findings rather than a limitation to their validity: Their help-seeking due to their sexual inclinations might have contributed to greater problems in acceptance than would be expected in a general population.

Even though we were able to analyze a larger sample than was available for the only one prior study on this issue, our sample size still impeded more sophisticated analyses like structural equation models, thus potentially overlooking further inter-associations of the constructs examined. This also leaves room for speculation on the clinical significance of the potential associations of acceptance of one’s sexual inclinations and fantasies concerning both offending behavior and distress. Such more sophisticated analyses might prove fruitful in future endeavors.

The measure applied in this preliminary study poses another limitation to its generalizability. Note that a formal validation of the measure remains missing, e.g., concerning measurement invariance in different populations, discriminant, convergent, or divergent validity. Validated measures aiming to address acceptance of one’s sexuality have been published for use in other sexual minorities since the inception of this study, like the Lesbian, Gay, and Bisexual Identity Scale, LGBIS (Mohr and Fassinger, 2000; Mohr and Kendra, 2011; Niepel et al., 2019), or the Self-Acceptance of Sexuality Inventory, SASI (Camp et al., 2022), with different application potential for the sample of pedohebephilic individuals.

Some constraints to the generalizability stem from our measure of the use of CSEM and the changes made to CSEM legislation over time. At the time of data assessment, imagery of lightly dressed and nude children was mostly unsanctioned in Germany. The formulation of the items assessing this type of imagery was, therefore, not well equipped for a classification according to current standards. Including and excluding the respective items changed some of the associations in the regression analysis, namely revealing an additional interaction effect of recent general offending on acceptance. Given the lack of specificity of the items, the relevance of this difference is impossible to gauge and should be addressed in future efforts.

## Conclusion

Our study yields first-time evidence for an association of acceptance of one’s pedohebephilic sexual inclinations and fantasies with psychological distress and its potential moderation by recent offending. With a positive association of acceptance of one’s sexual inclinations and fantasies with distress in non-offending pedohebephiles and an attenuated association in recent offending individuals, a differential therapeutic approach in both groups concerning acceptance of one’s sexual inclinations and fantasies might prove warranted. Given the psychometric problems and weak theoretical underpinnings of the instrument used in our study, further efforts will be needed to put these conclusions on firmer grounds, bearing in mind the ramifications of further aspects of the acceptance of one’s pedohebephilic sexuality, e.g., on health behavior, mental health, or psychosocial functioning.

## Data Availability

The datasets presented in this article are not readily available because of ethical concerns as the data contains highly sensitive and potentially self-incriminating information. Questions regarding the datasets should be directed to Anna Konrad (anna.konrad@charite.de).
