# Supplementary material for: Acceptance of sexual attraction and its link to psychological distress and sexual offending among pedohebephilic clients: results from a preliminary analysis
Source: Front Psychol. 2025 Jan 27;15:1463191. doi: 10.3389/fpsyg.2024.1463191 (PMC11841447; doi:10.3389/fpsyg.2024.1463191)
Supplement: Supplementary file 1 [file Data_Sheet_1.pdf]

Acceptance of sexual attraction and its link to psychological distress and sexual offending among pedohebephilic clients – results from a preliminary analysis

Supplementary material

Appendix A - Sociodemographic characteristics of the full sample and subsamples

	Full sample (*N* = 238) [a]					Subsample 1 (*n* = 197) [b]						Subsample 2 (*n* = 84) [c]
	Pedophilic (*n* = 118)	Hebephilic (*n* = 79)	Teleiophilic (*n* = 41)			Non-Offending (*n* = 31)	CSEM only (*n* = 130)	CSA only (*n* = 7)	Both offenses (*n* = 29)			
	n (%)	*n* (%)	*n* (%)	*F*(*df*)/ χ^2(*df*)	ω^2/*V*	*n* (%)	*n* (%)	*n* (%)	*n* (%)	*F*(*df*)	ω^2	*n* (%)
Education				2.84 (2)	.11					[F]		
Max 10 years	54 (46)	40 (51)	25 (61)			13 (42)	61 (47)	5 (71)	15 (52)			44 (52)
Min 11 years	64 (54)	39 (49)	16 (39)			18 (58)	69 (53)	2 (29)	14 (48)			40 (48)
Employment				2.83 (2)	.11					[F]		
No	43 (36)	22 (28)	10 (24)			10 (32)	38 (29)	2 (29)	15 (52)			33 (39)
Yes	74 (63)	56 (71)	31 (76)			21 (68)	90 (69)	5 (71)	14 (48)			51 (61)
No answer	1 (1)	1 (1)	–			–	2 (2)	–	–			
Relationship				5.43 (2) °	.15					[F]		59 (70)
No	80 (68)	47 (59)	19 (46)			22 (71)	79 (61)	3 (43)	23 (79)			25 (30)
yes	38 (32)	32 (41)	21 (51)			9 (29)	51 (39)	4 (57)	6 (21)			–
No answer	–	–	1 (2)			–	–	–	–			
Living alone				3.10 (2)	.12					[F]		47 (56)
Yes	66 (56)	35 (44)	23 (56)			18 (58)	63 (48)	3 (43)	17 (59)			37 (44)
No	50 (42)	43 (54)	17 (41)			13 (42)	65 (50)	4 (57)	11 (38)			–
No answer	2 (2)	1 (1)	1 (2)			–	2 (2)	–	1 (3)			
Children				3.42 (2)	.12					[F]		57 (58)
No	73 (62)	49 (62)	19 (46)			21 (67)	82 (63)	2 (29)	17 (59)			27 (32)
Yes	45 (38)	30 (38)	22 (54)			10 (32)	48 (37)	5 (71)	12 (41)			
Sexual age preference				–	–					[F]		
Pedophilic	118 (100)	–	–			20 (65)	76 (59)	5 (71)	17 (59)			56 (67)
Hebephilic	–	79 (100)	–			11 (36)	54 (42)	2 (29)	12 (41)			28 (33)
Teleiophilic	–	–	41 (100)			-	-	-	-			–
Exclusivity of sexual age preference										* [F]		
Exclusive	46 (39)	16 (20)	41 (100)			10 (32)	37 (29)	0 (0)	15 (52)			41 (49)
Non-exclusive	72 (61)	63 (80)	–			21 (68)	93 (72)	7 (100)	14 (48)			43 (51)
Preferred phenotypical sex				26.26 (4)***	.23					[F]		
Female	63 (53) [e]	47 (60)	40 (98) [d]			13 (42)	80 (62)	4 (57)	13 (45)			44 (52)
Male	36 (31)	21 (27)	0 (0) [e]			12 (39)	33 (25)	1 (14)	11 (38)			36 (43)
Both	19 (16)	11 (14)	1 (2)			6 (19)	17 (13)	2 (29)	5 (17)			4 (5)

	Full sample (N = 238) [a]					Subsample 1 (n = 197) [b]						Subsample 2 (n = 84) [c]
Recent offending behavior				***[F]						–	–	
Non-offending	20 (17)	11 (14)	28 (68) [d]			31 (100)	–	–	–			18 (21)
CSEM only	76 (64)	54 (68)	7 (17) [e]			–	130 (100)	–	–			49 (58)
CSA only	5 (4)	2 (3)	5 (12)			–	–	7 (100)	–			4 (5)
Mixed offending	17 (14)	12 (15)	1 (2)			–	–	–	29 (100)			13 (15)
Lifetime offending behavior				20.77 (2)***	.30					[F]		
Yes	77 (65) [d]	40 (51)	10 (24) [e]			18 (58)	76 (59)	6 (86)	17 (59)			78 (93)
No	41 (35) [e]	39 (49)	31 (76) [d]			13 (42)	54 (41)	1 (14)	12 (41)			6 (7)
Age				3.07 (2, 235)*	.02					1.26 (3, 24.55) [W]	.03	
M (*SD*)	36.0 (12.2) $_a$	39.6 (11.8) $_a$	40.5 (12.7) $_a$			40.3 (13.8)	36.1 (10.8)	37.1 (8.6)	40.5 (16.0)			38.3 (12.6)

Note. Group comparisons for age were calculated using one-way or Welch's ANOVA. Effect sizes are presented as ω^2 following the interpretation of (Kirk, 1996). Post hoc comparisons were calculated using Hochberg's GT2 test (Hochberg, 1974, 1975). Means in a row not sharing subscripts are significantly different from one another. Group comparison for categorical variables were calculated using Chi-square tests or in case of an expected cell frequency < 5 Fisher-Exact tests. For Chi-square tests Cramer's *V* is presented as effect size. Interpretation of Cramer's *V* followed J. Cohen (1988). Significant differences are bold.

° p < .1, * p < .05, ** p < .01, *** p < .001; [F] result of Fisher's exact test. [W] Welch's ANOVA

[a] Sample used for factor analysis (part 1) and sexual preference group comparisons (part 2a).

[b] Sample used for offending group comparisons (part 2b) and associations between acceptance and frequency of behavioral manifestations and sexual desire (part 3).

[c] Sample used for association of acceptance and psychological distress and recent offending behavior (part 4)

[d] Significantly more observed frequencies than expected (post-hoc chi-quadrat test, based on adjusted residuals, Bonferroni corrected alpha-level)

[e] Significantly less observed frequencies than expected (post-hoc chi-quadrat test, based on adjusted residuals, Bonferroni corrected alpha-level)

Appendix B – assessment of sexual behaviors and desire

Table B 1

Q-SENICA items used to rate recent CSEM-offending behavior. Item numbers are given in grey.

Type of CSEM	Q-SENICA items
Sexual assault minor<br>1b, 1c, 3c, 3b<br><br>OR	How often in the last 6 months did you consume (1) films, (2) images/photos with children/teenagers in/on which sexual acts are performed between an adult and a child/an adult and an adolescent for (a) masturbation, (b) stimulating pastime?
Explicit Act minor *(explicit posing minor + explicit sexual activity minor)*<br>1d, 3d, 8b, 8c, 10b, 10c<br><br>OR	How often in the last 6 months did you consume (1) films, (2) images/photos with children/teenagers in/on which sexual acts are performed between an adolescent and a child for (a) masturbation, (b) stimulating pastime?<br>How often in the last 6 months did you consume (1) films, (2) images/photos with children/adolescent in which sexual organs (e.g., buttocks, penis, vagina, breasts…) can be seen in detail and/or in which a child/adolescent masturbates for (a) masturbation, (b) stimulating pastime?
Erotic posing minor<br>15b, 15c, 17b, 17c, 19b, 19c, 21b, 21c	How often in the last 6 months did you consume (1) films, (2) images/photos of naked children/adolescents (e.g., on the beach, bathing, medically examined, or “posing” in front of a camera…) for (a) masturbation, (b) stimulating pastime?<br>How often in the last 6 months did you consume (1) films, (2) images/photos of lightly dressed children/adolescents (e.g., in underwear, gym shorts, swimming trunks, leotards, transparent clothing…) for (a) masturbation, (b) stimulating pastime?

Table B 2

Specific questions used to build the six variables which assess the frequency of the use of CSEM and frequency of sexual desire/behavior towards children. Item numbers are given in grey.

Variable	Items used from Q-SENICA, SBIMS, and FSEV
(1) Frequency of Use of Legal Imagery of Children (Q-SENICA items)<br>19b, 21b, 26b, 28b	How often in the last 6 months did you consume (1) films with, (2) images/photos of lightly dressed children (e.g., in underwear, gym shorts, swimming trunks, leotards, transparent clothing…) for (a) masturbation, (b) stimulating pastime?<br><br>How often in the last 6 months did you consume films with normally dressed children (e.g., children’s films, feature films, documentary films…) for (a) masturbation, (b) stimulating pastime?<br>How often in the last 6 months did you consume images/photos/portraits of dressed children (e.g., in magazines, on postcards, in illustrated books) for (a) masturbation, (b) stimulating pastime?
(2) Frequency of Use of Legal Imagery of Adolescents (Q-SENICA items)<br>19c, 21c, 26c, 28c	How often in the last 6 months did you consume (1) films with, (2) images/photos of lightly dressed adolescents (e.g., in underwear, gym shorts, swimming trunks, leotards, transparent clothing…) for (a) masturbation, (b) stimulating pastime?<br><br>How often in the last 6 months did you consume films with normally dressed adolescents (e.g., children’s films, feature films, documentary films…) for (a) masturbation, (b) stimulating pastime?

	How often in the last 6 months did you consume films with normally dressed adolescents (e.g., children’s films, feature films, documentary films…) for (a) masturbation, (b) stimulating pastime?
(3) Frequency of Use of Illegal Children Sexual Abuse Imagery (Q-SENICA items)<br>15b, 17b, 23b, 8b, 10b, 1b, 3b	How often in the last 6 months did you consume (1) films with, (2) images/photos of naked children (e.g., on the beach, bathing, medically examined, or posing in front of a camera) for (a) masturbation, (b) stimulating pastime?
	How often in the last 6 months did you consume images/photos of children who were dressed up or posed styled up (e.g., made up, in special clothing, school uniforms, sailor suits etc.) for (a) masturbation, (b) stimulating pastime?
	How often in the last 6 months did you consume (1) films, (2) images/photos with children in which sexual organs (e.g., buttocks, penis, vagina, breasts: : :) can be seen in detail and/or in which a child masturbates for (a) masturbation, (b) stimulating pastime?
	How often in the last 6 months did you consume (1) films, (2) images photos with children in/on which sexual acts are performed between an adult and a child for (a) masturbation, (b) stimulating pastime?
(4) Frequency of Use of Illegal Adolescent Sexual Abuse Imagery (Q-SENICA items)<br>1cb, 17c, 23c, 8c, 10c, 1c, 3c	How often in the last 6 months did you consume (1) films with, (2) images/photos of naked adolescents (e.g., on the beach, bathing, medically examined, or posing in front of a camera) for (a) masturbation, (b) stimulating pastime?
	How often in the last 6 months did you consume images/photos of adolescents who were dressed up or posed styled up (e.g., made up, in special clothing, school uniforms, sailor suits etc.) for (a) masturbation, (b) stimulating pastime?
	How often in the last 6 months did you consume (1) films, (2) images/photos with adolescents in which sexual organs (e.g., buttocks, penis, vagina, breasts…) can be seen in detail and/or in which a child masturbates for (a) masturbation, (b) stimulating pastime?
	How often in the last 6 months did you consume (1) films, (2) images photos with adolescents in/on which sexual acts are performed between an adult and an adolescent for (a) masturbation, (b) stimulating pastime?
(5) Frequency of Desire for Sexual Activities with Minors (Q-SEB items)	Within the past 12 months, how often do you have a desire for sex with intercourse with a child/adolescent?
	Within the past 12 months, how often do you have a desire for sex without intercourse with a child/adolescent?
	Within the past 12 months, how often do you have a desire for intimate body contact with a child/adolescent?
(6a) Frequency of Sexual Activities with Minors within the past 6 months (SBIMS items)	Within the past six months, how frequently did you engage in sexual contact with a prepubescent/pubescent child such as sexual intercourse (vaginal, anal, oral), fondling or kissing of genitals or mutual masturbation in real life?
	Within the past six months, how frequently did you engage in sexual interactions with a prepubescent/pubescent child such as sexual talks, showing pornography, or recording pornographic images of children?
	Within the past six months, how frequently did you engage in physical contact with a prepubescent/pubescent child such as washing, caressing or help putting on cream in real life?
(6a) Frequency of Sexual Activities with Minors within the past year (Q-SEB items)	How often do you normally have intercourse with a child/adolescent?
	How often do you normally have sex without intercourse with a child/adolescent?
	How often do you normally have intimate body contact with a child/adolescent?

Table B 3

Inter-item correlations of the items for frequency of the use of abuse/exploitation material of minors and frequency of sexual desire/behavior toward minors

Item	1	2	3	4	5	6a
Frequency of…						
(1) Use of Legal Imagery of Children						
(2) Use of Legal Imagery of Adolescents	.66***					
(3) Use of Illegal Child Sexual Abuse Imagery	.60***	.31***				
(4) Use of Illegal Adolescent Sexual Abuse Imagery	.28***	.49***	.51***			
(5) Desire for Sexual Activities with Minors	.31***	.27***	.32***	.22**		
(6a) Sexual Activities with Minors (SBIMS) [a]	-.04	.00	-.06	.03	.12	
(6b) Sexual Activities with Minors (Q-SEB) [b]	.04	.09	-.07	-.06	.30***	.31***

Note. Correlation coefficient = Pearson. (1)–(4), (6a): Frequency within the past 6 months. (5), (6b): Frequency within the past 12 months. [a] Amalgamated items of the SBIMS to assess Frequency of Sexual Activity with Minors within the past 6 months. [b] Amalgamated items of the Q-SEB to assess Frequency of Sexual Activity with Minors within the past 12 months.

* $p < .05$. ** $p < .01$. *** $p < .001$.

Appendix C - Psychometric and exploratory factor analyses of the IASI

Item and reliability analysis of the 15 IASI items including inspection of item distribution, measures of centrality and variability, item difficulty, discriminatory power, and internal consistency (Cronbach's α) were computed in the full sample (*N* = 238). Results of α = .80 or more were considered adequate (Nunnally, 1978). We defined extreme univariate skewness as ≥ 2.0 and kurtosis of ≥ 7.0 and evaluated multivariate skew and kurtosis with Mardia's estimates (Mardia, 1970; Watkins, 2018). Bartlett's Test of Sphericity was used to test that the correlation matrix was not random. The Kaiser-Meyer-Olkin measure (KMO; (Kaiser & Rice, 1974)) was used to test for sampling adequacy. Values of the measure were deemed as follows: < .5 = *"barely acceptable"*, .5 – .7 = "*mediocre*", .7 – .8 = "*good*", .8 – .9 = "*great*"; > .9 = "*superb"* (Hutcheson & Sofroniou, 1999). To rule out multicollinearity, a determinant of of the *R*-matrix of *R* > .00001 was set (Field, 2013).

We chose common factor analysis with iterated principal axis method with initial communalities estimated by squared multiple correlations due to its relative tolerance of non-normality and demonstrated ability to recover weak factors (Briggs & MacCallum, 2003; Guttman, 1956). Number of factors to be retained were determined using the visual scree plot, parallel test with 100 repetitions, minimum average partials (MAP), and the interpretability of the solution (Cattell, 1966; Horn, 1965; Velicer, 1976). As we assumed correlated factors, we compared the results of the oblique rotation algorithms "oblimin" and "promax". Pattern coefficients ≥ .335 were set to be both statistically and practically significant (i.e. salient) (Watkins, 2018). Complex loadings that were salient on more than one factor were rejected to honour the simple structure principle. Theoretically meaningful factors with a minimum of three salient pattern coefficients, internal consistency reliability ≥ .70 were considered adequate (Watkins, 2018).

Psychometric analyses

A translation of the items can be found in table 1. The initial internal consistency of the overall scale was adequate (Cronbach's α = .88, 95% CI [.85, .90]), with mean inter-item correlations of .30. Items 12 and 13 showed discriminatory power < .2, high means and low standard deviations compared to the other items, left-skewed distributions, and high item difficulties. Items 14 and 5 also showed

corrected item-total correlations (discriminatory power) smaller than .3. Item 10 showed a left-skewed distribution as well as a high mean and item difficulty compared to the other items and discriminatory power just above .3. Descriptive statistics and distributions of the items are given in Appendix C, Figure C 1, Table C 1. Dropping items 5, 10, and 12 through 14 only had a marginal impact on the internal consistency. Since the initial internal consistency was adequate, no item was excluded from the following exploratory factor analysis (EFA). Due to the ordinal nature of the IASI items, the presence of one extreme univariate skew, as well as a statistically significant Mardia's multivariate skew ($p < .001$), we used a polychoric correlation matrix as input for the EFA. Here, items 5, 10, 12, 13, and 14 showed only low inter-item correlations (see Table C 2).

Exploratory factor analysis

Data were deemed suitable for a Principal Axis Factor Analysis (PAF) with an overall Kaiser-Meyer-Olkin measure of .88, KMO values for individual items ranging from .58 to .93 (see Table C 3) and a significant Bartlett's test of sphericity ($\chi^2(105) = 2405.05$, $p < .001$). The determinant of the correlation matrix of .00003 indicated no mulicollinearity. MAP suggested two factors, parallel analysis five and the scree-plot four factors (see Figure 1). Given these diverging results, we examined the five-factor to two-factor solutions sequentially.

The five- and four-factor solutions accounted for a respective 66.33% and 62.54% of variance after oblimin rotation. Complex loadings were determined for three items in the five-factor solution (items 8, 2, 10) and for one item in the four-factor solution (item 13). Both solutions bore two factors that did not reach the minimum of three salient pattern coefficients and were deemed underdeterminated. The five- and four-factor solutions were thus deemed inadequate.

The three-factor solution accounted for 58.51% of variance after oblimin rotation and comprised five items with complex loadings, namely items 2, 7, 12, 15 loading saliently on factors 1 and 2 and item 10 loading salienty on factors 1 and 3. Rejecting these items led to underdetermination of factor 3 with only two salient pattern coeffiencients (item 5 and 13). Factor 2, comprising items 8, 1, and 14, showed only questionable internal consistency (α = .63, 95% CI [.53,

.70]) while factor 1 (items 3, 4, 11, 6, 9) showed excellent internal consistency (α = .92, 95% CI [.90, .93]).

The two-factor solution accounted for 53.76% of variance after oblimin rotation. There was one item (8) with a complex loading and one item (10) that did not load significantly on either factor. Rejection of those two items led to nine items loading saliently on factor 1 and four items loading saliently on factor 2. The internal consistency of factor 1 was excellent (α = .92, 95% CI [.90, .93]), while it was poor for factor 2 (α = .47, 95% CI [.35, .57]). Oblimin and promax rotation yielded comparable results. The structure matrix and pattern matrix produced similar coefficients (see Table C 4, Table C 5). Factors 1 and 2 showed a low medium correlation (r = .29).

Figure C 1

Item distribution of the 15 IASI items in the full sample (N = 238)

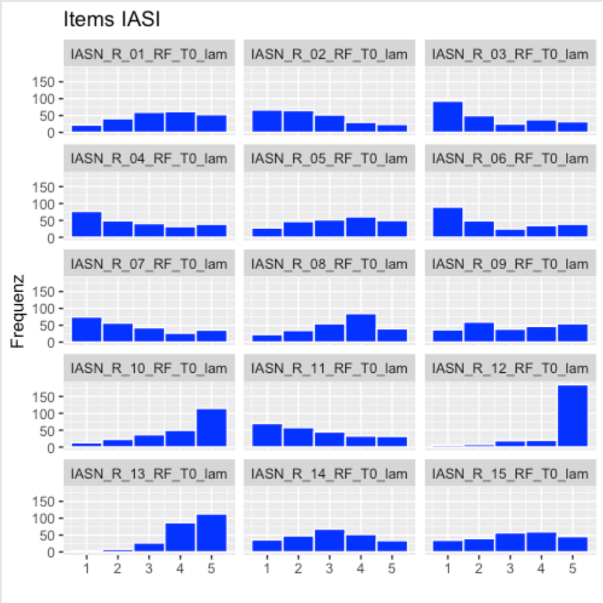

Figure C 2

Parallel analysis and scree plot of the Principal Axis Factor Analysis based on a polychoric correlation matrix.

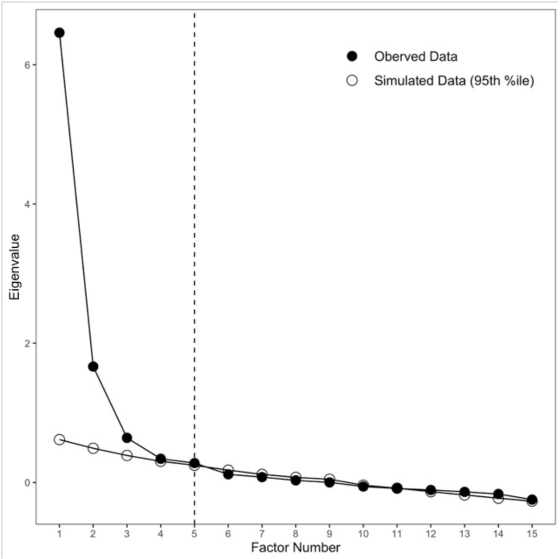

Note. The dashed vertical line indicates the max number of factors to be retained suggested by the parallel analysis with 100 repetitions.

Table C 1

Item statistics of the IASI (N = 238)

Item	*M*	*SD*	Skew	Kurtosis	Difficulty (P_i)	Corrected item-scale correlation (discriminatory power) $R_{it,c}$	Raw α when item dropped
01 (inv) – forbid sexual fantasies	3.35	1.26	-0.28	-0.96	58.72	.64	.87
02 – enjoy sexual fantasies	2.49	1.30	0.50	-0.84	37.29	.72	.86
03 (inv) – hate sexual inclination	2.44	1.47	0.54	-1.20	35.92	.71	.86
04 (inv) – cannot accept sexual inclination	2.61	1.47	0.39	-1.25	40.23	.76	.86
05 – inclination integral to personality	3.24	1.31	-0.21	-1.11	56.09	.28	.88
06 (inv) – reject sexual inclination	2.51	1.51	0.48	-1.29	37.82	.74	.86
07 (inv) – resist sexual fantasies	2.55	1.42	0.49	-1.09	38.66	.56	.87
08 – allow sexual fantasies	3.35	1.20	-0.45	-0.72	58.82	.63	.87
09 (inv) – reject myself for inclination	3.10	1.41	-0.01	-1.36	52.82	.61	.87
10 (inv) – sexual fantasies unrelated to me	3.97	1.23	-0.95	-0.24	74.26	.31	.88
11 (inv) – sexual fantasies scare me	2.58	1.39	0.42	-1.09	39.39	.68	.86
12 (inv) – have no sexual fantasies	4.55	0.96	-2.22	4.12	88.87	.10	.89
13 – aware of sexual inclination	4.26	0.88	-1.26	1.54	81.41	.16	.88
14 – embellish sexual fantasies	3.00	1.27	-0.02	-1.01	50.00	.20	.88
15 (inv) – resist sexual fantasies	3.18	1.33	-0.21	-1.10	54.41	.72	.86

Note. The overall mean was 3.10 (*SD* = 0.79).

Table C 2

Inter-item correlation matrix of the IASI items (N = 238)

Item	01	02	03	04	05	06	07	08	09	10	11	12	13	14	15
01 – forbid sexual fantasies	1														
02 – enjoy sexual fantasies	.66	1													
03- hate sexual inclination	.54	.67	1												
04 – cannot accept sexual inclination	.54	.70	.82	1											
05 – inclination integral to personality	**.17**	**.22**	**.16**	**.22**	1										
06 – reject sexual inclination	.54	.70	.85	.84	**.22**	1									
07 – resist sexual fantasies	.57	.47	.53	.52	**.21**	.55	1								
08 – allow sexual fantasies	.63	.62	.40	.45	.30	.51	.45	1							
09 – reject myself for inclination	.49	.55	.78	.76	**-.04**	.69	.45	.30	1						
10 – sexual fantasies unrelated to me	**.19**	**.19**	**.23**	.34	.36	**.23**	**.22**	.31	**.23**	1					
11 – sexual fantasies scare me	.54	.64	.75	.73	**.11**	.68	.51	.39	.69	.37	1				
12 – have no sexual fantasies	**.18**	**.10**	**-.09**	**.02**	**.18**	**-.06**	**.10**	.42	**-.04**	**.16**	**-.10**	1			
13 – aware of sexual inclination	**.10**	**.02**	**.05**	**.09**	.31	**.02**	**-.02**	**.27**	**.07**	**.25**	**.08**	.33	1		
14 – embellish sexual fantasies	**.19**	.23	**.04**	**.09**	**.21**	**.07**	**.11**	.39	**.01**	**.13**	**.00**	.45	**.17**	1	
15 – resist sexual fantasies	.70	.67	.62	.63	**.21**	.64	.58	.68	.51	.32	.70	**.16**	**.10**	**.23**	1

Note. Correlations < .30 are bold. Measure of correlation = polychoric correlation coefficient

Table C 3

Measure of Adequacy for each of the 15 items of the IASI (N = 238)

Item	KMO
01 – forbid sexual fantasies	.92
02 – enjoy sexual fantasies	.91
03 – hate sexual inclination	.91
04 – cannot accept sexual inclination	.92
05 – inclination integral to personality	.66
06 – reject sexual inclination	.89
07 – resist sexual fantasies	.93
08 – allow sexual fantasies	.84
09 – reject myself for inclination	.89
10 – sexual fantasies unrelated to me	.77
11 – sexual fantasies scare me	.90
12 – have no sexual fantasies	.69
13 – aware of sexual inclination	.58
14 – embellish sexual fantasies	.81
15 – resist sexual fantasies	.91

Note. KMO = Kaiser-Meyer-Olkin measure of sampling adequacy

Table C 4

Structure matrix of the correlations between factors of the two-factor solution and the 15 IASI items (N = 238)

Item	Correlation between item and factor	
	Factor 1	Factor 2
3 – Ich hasse meine sexuelle Neigung. [a]<br>*I hate my sexual inclination.*	.90	.15
4 – Ich kann meine sexuelle Neigung nicht akzeptieren. [a]<br>*I cannot accept my sexual inclination.*	.89	.27
6 – Ich lehne meine sexuelle Neigung ab. [a]<br>*I reject my sexual inclination.*	.88	.24
11 – Meine sexuellen Fantasien erschrecken mich. [a]<br>*My sexual fantasies scare me.*	.84	.19
9 – Ich lehne mich ab, weil ich diese sexuelle Neigung habe. [a]<br>*I reject myself because I have this sexual inclination.*	.79	.09
2 – Ich kann meine sexuellen Fantasien ohne schlechtes Gewissen genießen.<br>*I can enjoy my sexual fantasies without a bad conscience.*	.78	.43
15 – Ich wehre mich gegen meine sexuellen Fantasien. [a]<br>*I resist my sexual fantasies.*	.77	.53
1 – Ich verbiete mir meine sexuellen Fantasien. [a]<br>*I forbid myself my sexual fantasies.*	.68	.49
7 – Gegen unangenehme sexuelle Fantasien muss man sich wehren. [a]<br>*One must resist disagreeable sexual fantasies.*	.62	.34
8 – Ich lasse meine sexuellen Fantasien zu.<br>*I allow myself my sexual fantasies.*	.55	.78
12 – Ich habe keine sexuellen Fantasien. [a]<br>*I have no sexual fantasies.*	-.03	.64
14 – Ich schmücke meine sexuellen Fantasien gerne aus.<br>*I like to embellish my sexual fantasies.*	.08	.54
5 – Meine Neigung ist ein fester Bestandteil meiner Persönlichkeit.<br>*My inclination is an integral part of my personality.*	.18	.43
13 – Ich bin mir meiner sexuellen Neigung bewusst.<br>*I am aware of my sexual inclination.*	.05	.39
10 – Meine sexuellen Fantasien haben nichts mit mir zu tun. [a]<br>*My sexual fantasies have nothing to do with me.*	.31	.35

Note. Extraction method: Principal axis factor analysis. Rotation method: oblimin rotation.

[a] Inverted items.

Table C 5

Matrix of the pattern coefficients of the two-factor solution for the 15 IASI items (N = 238).

Item	Rotated factor loadings		h^2
	Factor 1	Factor 2	
3 – Ich hasse meine sexuelle Neigung. [a]<br>*I hate my sexual inclination.*	**.94**	-.12	.83
6 – Ich lehne meine sexuelle Neigung ab. [a]<br>*I reject my sexual inclination.*	**.89**	-.02	.78
4 – Ich kann meine sexuelle Neigung nicht akzeptieren. [a]<br>*I cannot accept my sexual inclination.*	**.88**	.01	.79
11 – Meine sexuellen Fantasien erschrecken mich. [a]<br>*My sexual fantasies scare me.*	**.86**	-.06	.71
9 – Ich lehne mich ab, weil ich diese sexuelle Neigung habe. [a]<br>*I reject myself because I have this sexual inclination.*	**.83**	-.15	.64
2 – Ich kann meine sexuellen Fantasien ohne schlechtes Gewissen genießen.<br>*I can enjoy my sexual fantasies without a bad conscience.*	**.72**	.22	.65
15 – Ich wehre mich gegen meine sexuellen Fantasien. [a]<br>*I resist my sexual fantasies.*	**.67**	.33	.70
1 – Ich verbiete mir meine sexuellen Fantasien. [a]<br>*I forbid myself my sexual fantasies.*	**.59**	.32	.55
7 – Gegen unangenehme sexuelle Fantasien muss man sich wehren. [a]<br>*One must resist disagreeable sexual fantasies.*	**.57**	.17	.41
12 – Ich habe keine sexuellen Fantasien. [a]<br>*I have no sexual fantasies.*	-.23	**.71**	.46
8 – Ich lasse meine sexuellen Fantasien zu.<br>*I allow myself my sexual fantasies.*	**.35**	**.68**	.73
14 – Ich schmücke meine sexuellen Fantasien gerne aus.<br>*I like to embellish my sexual fantasies.*	-.08	**.57**	.30
13 – Ich bin mir meiner sexuellen Neigung bewusst.<br>*I am aware of my sexual inclination.*	-.07	**.41**	.16
5 – Meine Neigung ist ein fester Bestandteil meiner Persönlichkeit.<br>*My inclination is an integral part of my personality.*	.06	**.41**	.19
10 – Meine sexuellen Fantasien haben nichts mit mir zu tun. [a]<br>*My sexual fantasies have nothing to do with me.*	.22	.29	.17
Eigenvalues	5.90	2.17	
Proportional variance	.39	.14	
Cumulative variance	.39	.54	
Cronbach's α [95% CI]	.92<br>[.90, .93]	.47<br>[.35, .57]	

Note. Extraction method: Principal axis factor analysis. Rotation method: oblimin. h^2 = communalities. Factor loadings ≥ .335 are bold.
[a] Items inverted.

Table C 6.

Reliability analysis statistics for the two subscales of the IASI based on the extracted factors (N = 238)

Item	Corrected item-scale correlation (discriminatory power) $r_{it(i)}$	Raw α when item dropped
	Factor 1 (α = .92)	
03 (inv)	.79	.91
06 (inv)	.79	.91
04 (inv)	.81	.91
11 (inv)	.75	.91
09 (inv)	.70	.91
02	.72	.91
15 (inv)	.70	.91
01 (inv)	.63	.92
07 (inv)	.56	.92
	Factor 2 (α = .47)	
12 (inv)	.39	.29
14	.38	.30
13	.41	.27
05	.42	.26

Note. Inv = inverted items.

Appendix D – Sensitivity Analysis CSEM offending behavior

The items from the Q-SENICA of "lightly dressed minors (e.g., in underwear, gym shorts, swimming trunks, leotards, transparent clothing…) reflect imagery that by today's legislation may comprise illegal as well as legal materials. For our main analyses, we chose to classify positive responses on these items as representing the use of "erotic posing" imagery and hence as illegal. To assess the potential influence of this decision, we re-ran the comparison of groups separated by recent offending and the regression analysis using offense variables classifying responses as legal behaviors.

Prevalences

	**Subsample 1 (n = 197)**		**Subsample 2 (n = 84)**	
	original	„lightly dressed minors" as legal imagery	original	„lightly dressed minors" as legal imagery
Non-offending	31	40	18	21
CSEM only	130	121	49	46
CSA only	7	8	4	4
Both offenses	29	28	13	13
Recent CSEM offending	159	149	62	59
Recent CSA offending	36	36	17	17

Group comparison: Acceptance by recent offense behavior

Median values: non-offending= 22; CSEM_only = 21; CSA_only = 22; mixed = 23.5

$H(3) = 6.217$, p = .102; $\varepsilon = 0.03$ [0.007, 0.103]

Regression: General offending behavior

Model without interaction: ($F(2, 81) = 6.95$, $p = .002$)
Model with interaction: $F(3,80) = 7.85$, $p < .001$
Comparison of both models: ($F(1, 80) = 8.39$, $p = .005$) → added interaction significantly improves the model

- General offending significantly associated with acceptance ($p = .028$)
- Distress significantly associated with acceptance ($p < .001$)
- Interaction significantly associated with acceptance ($p = .005$)
    - Simple slope offending: $b = -3.40$ [-6.66, -0.15], $p = .041$
    - Simple slope non-offending: $b = -13.72$, $p < .001$
    - **Greater effect of distress on acceptance in recent non-offending individuals than offending**

Coefficients	Step 1 (without interaction)				Step 2 (with interaction)			
		95% CI				95% CI		
	b	LL	UL	SE	b	LL	UL	SE
Constant	27.53***	23.49	31.58	2.03	33.74***	27.98	39.50	2.89
Offending1	0.70	-3.28	4.67	2.00	-7.91*	-14.94	-0.88	3.53

GSI	-5.58***	-8.59	-2.56	1.52	-13.72***	-20.02	-7.42	3.17
Offending1 x GSI	–	–	–	–	10.32**	3.23	17.41	3.56
R2				.15**				.23***
Adjusted R2				.13				.20

Note. Dependent variable: Sum score of the IASI-9 "Acceptance of one's sexual fantasies and inclinations". Offending variable coded as 0 = Non-Offending, 1 = Offending. GSI = Global Severity Index of the *Brief Symptom Inventory.*
* *p* <.05 **, *p* < .01, *** *p* <.001

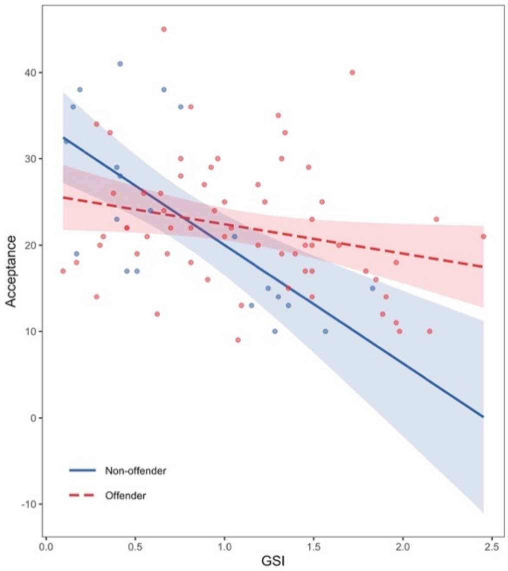

Appendix E – Sensitivity analysis for SBIMS item 3 "non-sexual contact"

Item 3 of the SBIMS is phrased: "In past 6 months Frequency of occurrence of non-sexual interactions with a prebubescent/pubenscent child, such as washing, caressing, or help putting on cream? (in real life)". From a clinical standpoint, this item covers abusive behaviors that sexual offending individuals may trivialize as "non-sexual". At the same time, these "non-sexual" behaviors may not reflect abusive behaviors at all. We thus chose to conduct a sensitivity analysis to ascertain the influence of this item on our results.

Prevalences

	**Subsample 1 (n = 197)**		**Subsample 2 (n = 84)**	
	Item 3 as CSA	Item 3 not as CSA	Item 3 as CSA	Item 3 not as CSA
Non-offending	31	31	18	18
CSEM only	130	134	49	51
CSA only	7	7	4	4
mixed	29	25	13	11
CSEM_offending	159	156	62	62
CSA_offending (SBIMS)	36	32	17	15

Group comparison of acceptance by recent offense behavior

Median values: non-offending = 19; CSEM_only = 21.5; CSA_only = 22; mixed = 25

$H(3) = 7.358$, p = .061; $\varepsilon = 0.04$ [0.01, 0.11]

Correlation between acceptance and frequency of sexual activities with minors in the last six months

Variable	*Mdn*	Range	*rho*	FDR adjusted p_{BH}	*n*
(6a) … Sexual Activities with Minors (SBIMS)	1	1-5	.195	.041	197

Note. Correlation coefficients computed as Spearman's rho. 6a: Frequency of sexual activity with minors within the past 6 months from amalgamated items 1 and 2 of the SBIMS. Acceptance measured with the IASI-9 = Nine item version retained after exploratory factor analysis (see supplemental material C for details) of the Inventory for the Acceptance of Sexual Preference.

Regression: General offending behavior

Model without interaction: (F(2, 81) = 7.02, p = .002)
Model with interaction: F(3,80) = 6.672, p < .001
Comparison of both models: (F(1, 80) = 5.25, p = .025) → added interaction significantly improves the model

- Distress significantly associated with acceptance (p < .001)
- Interaction significantly associated with acceptance (p = .024)
    - Simple slope offending b = -4.02 [-7.24, -0.80], p = .015
    - Simple slope non-offending: b = -12.73, p < .001
    - **Greater effect of distress in recent non-offending individuals than offending**

Coefficients	Step 1 (without interaction)				Step 2 (with interaction)			
		95% CI				95% CI		
	b	*LL*	*UL*	*SE*	*b*	*LL*	*UL*	*SE*
Constant	27.28***	23.01	31.55	2.15	32.79***	26.45	39.13	3.19
Offending1	1.02	-3.13	5.17	2.09	-6.20	-13.67	1.26	3.75
GSI	-5.60***	-8.59	-2.61	1.50	-12.73***	-19.57	-5.89	3.44
Offending1 x GSI	–	–	–	–	8.71*	1.14	16.26	3.56
R^2				.15**				.20***
Adjusted R^2				.13				.17

Note. Dependent variable: Sum score of the IASI-9. Offending variable coded as 0 = Non-Offending, 1 = Offending. GSI = Global Severity Index of the *Brief Symptom Inventory.* Factor 1: "Absence of rejection of one's sexual fantasies and inclinations".
* p <.05 **, p < .01, *** p <.001
